# Value of C-Reactive Protein in Predicting Left Ventricular Remodelling in Patients with a First ST-Segment Elevation Myocardial Infarction

**DOI:** 10.1155/2012/250867

**Published:** 2012-09-02

**Authors:** Iwona Swiatkiewicz, Marek Kozinski, Przemyslaw Magielski, Tomasz Fabiszak, Adam Sukiennik, Eliano Pio Navarese, Grazyna Odrowaz-Sypniewska, Jacek Kubica

**Affiliations:** ^1^Department of Cardiology and Internal Medicine, Collegium Medicum, Nicolaus Copernicus University, 9 Sklodowskiej-Curie Street, 85-094 Bydgoszcz, Poland; ^2^Department of Laboratory Diagnostics, Collegium Medicum, Nicolaus Copernicus University, 9 Sklodowskiej-Curie Street, 85-094 Bydgoszcz, Poland

## Abstract

*Objective*. To assess the value of C-reactive protein (CRP) in predicting postinfarct left ventricular remodelling (LVR). *Methods.* We measured in-hospital plasma CRP concentrations in patients with a first ST-segment elevation myocardial infarction (STEMI). *Results*. LVR was present at 6 months in 27.8% of 198 patients. CRP concentration rose during the first 24 h, mainly in LVR group. The prevalence of LVR was higher in patients from the highest quartile of CRP concentrations at 24 h as compared to those from any other quartile (odds ratio (OR) 3.48, 95% confidence interval (95% CI) 1.76–6.88). Multivariate analysis identified CRP concentration at 24 h (OR for a 10 mg/L increase 1.29, 95% CI 1.04–1.60), B-type natriuretic peptide at discharge (OR for a 100 pg/mL increase 1.21, 95% CI 1.05–1.39), body mass index (OR for a 1 kg/m^2^ increase 1.10, 95% CI 1.01–1.21), and left ventricular end-diastolic volume (OR for a 1 mL increase 0.98, 95% CI 0.96-0.99) as independent predictors of LVR. The ROC analysis revealed a limited discriminative value of CRP (area under the curve 0.61; 95% CI 0.54–0.68) in terms of LVR prediction. *Conclusions*. Measurement of CRP concentration at 24 h after admission possesses a significant but modest value in predicting LVR after a first STEMI.

## 1. Introduction

Left ventricular remodelling (LVR) often complicates acute ST-segment elevation myocardial infarction (STEMI) [1, 2]. The acute loss of myocardium results in an abrupt increase in loading conditions that induces LVR, the process by which ventricular size, shape, and function are regulated by mechanical, neurohormonal, and genetic factors [3]. Postinfarct LVR leads to a progressive rise in systolic and diastolic left ventricular volumes, distortion of ventricular shape and mural hypertrophy, in the weeks and months after STEMI [3]. It has been identified as an important marker of poor prognosis, linked with excessive cardiovascular mortality and risk of heart failure [1–4]. The major determinants of LVR after STEMI include infarct size, anterior location of infarction, and late or unsuccessful reperfusion therapy both at the epicardial artery level and at the microvascular level, transmurality of the infarct and extent of myocardial stunning [3–7].

Acute myocardial infarction (MI) is associated with a systemic inflammatory response with augmented production of nonspecific plasma acute-phase proteins, including C-reactive protein (CRP) [8]. The increase in plasma CRP concentration in the course of acute MI takes place in the first hours since the onset of symptoms and peaks approximately on day 2 [8]. The cytokines released in the progress of myocardial damage influence reactions affecting the expansion of necrosis and scar formation such as cell growth and migration as well as repairing processes [9, 10]. The tissue inflammatory response is not limited to the area of necrosis, but it extends to the intact portions of myocardium, where the expression of inflammatory cytokines continues to be modified. There is experimental evidence that an exaggerated inflammatory response might also promote tissue injury, contributing to chronic cardiac dilatation [10, 11].

Elevated plasma CRP concentration is associated with an unfavourable outcome in acute MI and allows clinicians to stratify patients in terms of their risk of death and heart failure [12, 13]. Additionally, evaluation of CRP concentration in patients with acute coronary syndromes provides prognostic information independent from the classical risk factors and enhances the value of well-established risk scores [13]. However, there is limited and partially conflicting data regarding the possible link between CRP and the development of LVR in humans [14–16]. Moreover, quantification of the potential additive effect of plasma CRP concentration besides well-established LVR predictors warrants further investigations.

We therefore set out to assess the value of in-hospital measurement of plasma CRP concentration in predicting the long-term risk of LVR in patients with a first STEMI, treated with primary percutaneous coronary intervention (pPCI).

## 2. Material and Methods

### 2.1. Study Design and Patient Characteristics

This study was designed as a single-center prospective observational cohort trial in the setting of first STEMI treated with pPCI, with 221 consecutive patients enrolled in the Department of Cardiology and Internal Medicine of Antoni Jurasz University Hospital in Bydgoszcz between 25th November 2005 and 27th November 2008. The inclusion criteria were as follows: (i) typical stenocardial chest pain of at least 30 minutes' duration, (ii) onset of symptoms <12 h before hospital admission, and (iii) electrocardiographic features of acute STEMI (ST-segment elevation ≥0,1 mV or ≥0,2 mV in at least 2 continuous limb or precordial leads, respectively). The exclusion criteria included (i) prior coronary revascularization, (ii) cardiogenic shock on admission, (iii) heart failure (New York Heart Association class III or IV), (iv) bundle branch block, (v) permanent atrial fibrillation, (vi) haemodynamically significant valvular heart disease, (vii) primary cardiomyopathy, (viii) severe arterial hypertension, (ix) creatinine concentration >176.8 mmol/L, (x) the presence of features suggestive of an active inflammatory process on admission, and (xi) therapy with steroids, immunosuppressive agents, and nonsteroidal anti-inflammatory drugs (excluding low doses of aspirin).

The study endpoint was LVR which was defined according to the previously validated criterion [2, 4], as a relative >20% increase in end-diastolic left ventricular volume (LVEDV) by echocardiography seen at 6-month followup compared with the baseline at discharge. The patients were divided, according to the presence of LVR at 6 months after STEMI into subgroups with (LVR group) and without (no LVR group) LVR.

Approval from the local Bioethics Committee at Collegium Medicum in Bydgoszcz was obtained. All patients gave their written, voluntary, informed consent for participation in the study.

### 2.2. Pharmacotherapy

At the first contact with health care providers immediately after establishing the diagnosis of STEMI all patients were pretreated with an intravenous bolus of unfractionated heparin (70 IU/kg, up to 5000 IU) and oral loading doses of clopidogrel (600 mg) and aspirin (300 mg). At the catheterization laboratory, second dose of unfractionated heparin was administered intraarterially in a weight-adjusted manner (up to 100 IU/kg) or under activated clotting time guidance (to the target range of 200–250 seconds) when abciximab was intended. Abciximab was given at the discretion of the invasive cardiologist. Throughout the study period clopidogrel and aspirin 75 mg q.d. were continued in all patients. Concomitant medications in the majority of patients included perindopril and long-acting metoprolol in doses adjusted for resting heart rate and blood pressure, and simvastatin 40 mg q.d. (Table 1). Additionally, at hospital discharge 12 (6.1%) patients were treated with spironolactone while 11 (5.6%) participants received nonpotassium-sparing diuretics.

### 2.3. Coronarography and pPCI

Coronarography and pPCI were performed using a standard femoral approach. The use of aspiration thrombectomy during the intervention was left to the operator's discretion. Intracoronary stents were routinely implanted. Coronary artery stenosis was measured with quantitative coronary angiography. Epicardial coronary flow was assessed according to the TIMI (Thrombolysis in Myocardial Infarction) score and TFC (TIMI frame count), and myocardial perfusion according to the TMPG (TIMI Myocardial Perfusion Grade).

### 2.4. Echocardiography

Transthoracic echocardiographic recordings employing the Doppler technique were acquired at hospital discharge and 6 months after STEMI using a Philips SONOS 7500 Ultrasound System, in accordance to the protocol recommended by The American Society of Echocardiography [17]. Echocardiographic recordings were assessed offline by two independent experienced echocardiographers blinded to the values of biomarker measurement. Measurements are reported as an average of three consecutive cardiac cycles. The corresponding echocardiographic results obtained by each echocardiographer were averaged. The inter- and intraobserver coefficients of variation for left ventricular end-diastolic volume (LVEDV) assessed in first 50 patients were below 5.0% and below 2.5%, respectively.

Left ventricular (LV) volumes and left ventricular ejection fraction (LVEF), a marker of global LV systolic function, were calculated using the modified Simpson rule [17]. Wall motion score index (WMSI), reflecting regional LV systolic function, was derived as a sum of all scores divided by the number of segments visualized, implementing the 16-segment model of LV segmentation and assigning a score from 1 point (normal) to 4 points (dyskinesia), respectively [18]. Left ventricular mass (LVM) was calculated according to the Devereux formula [19]. Measurements of peak mitral annular velocities were obtained for four basal segments of LV (septal, lateral, inferior, and anterior) using pulsed tissue Doppler echocardiography with the Doppler gate targeted at the junction of LV walls with the mitral annulus in 4- and 2-chamber views. Average peak systolic mitral annular velocity (*S*′), a marker of longitudinal LV systolic function, was obtained. Diastolic LV function was assessed using pulsed Doppler echocardiography and pulsed tissue Doppler echocardiography by measurements of peak velocity transmitral flow in the early phase (*E*) and during atrial systole (*A*) to obtain the *E*/*A* ratio, deceleration time of early transmitral flow and the *E*/*E*′ ratio, where *E*′ is the average peak early diastolic mitral annular velocity.

### 2.5. Blood Sampling and Laboratory Analyses

Peripheral venous blood samples were collected using ethylenediaminetetraacetic acid tubes. When centrifuged, the plasma was stored at −80°C until analyzed.

CRP plasma concentrations were measured with an ultrasensitive latex immunoassay (CRP Vario test, analyzer: ARCHITECT ci 8200, ABBOTT) on admission, 24 h after admission and at discharge. B-type natriuretic peptide (BNP) plasma concentration was evaluated with a chemiluminescent microparticle immunoassay (analyzer: ARCHITECT ci 8200, ABBOTT) on admission and at discharge. The limits of detection for CRP and BNP were 0.1 mg/L and 10 pg/L, respectively. The intraassay coefficients of variation were below 2.0% for CRP and below 5.0% for BNP, while the inter-assay coefficients of variation were below 1.0% for CRP and below 5.0% for BNP, respectively.

Additional biochemical measurements were performed using the following methods: blood glucose—hexokinase enzymatic glucose assay (Abbott, Wiesbaden, Germany); creatine kinase MB isoenzyme—CK-MB liquid immunological test assay (Sentinel Diagnostics, Milan, Italy); troponin I—chemiluminescence immunoassay STAT Troponin-I Architect System (Abbott, Middletown, OH, USA); creatinine—spectrophotometric creatinine assay (Abbott, Wiesbaden, Germany); total cholesterol, high-density-lipoprotein cholesterol and triglycerides—enzymatic cholesterol, ultrahigh-density-lipoprotein, and triglyceride assays (Abbott, Wiesbaden, Germany), respectively. Low-density-lipoprotein cholesterol concentration was calculated according to the Friedewald formula.

### 2.6. Statistical Analysis

Due to major advances in STEMI management resulting in improved survival and lower prevalence of postinfarct LVR along with reductions in mean CRP values in STEMI patients in recent years, we decided to perform an internal pilot study of first 50 patients for estimating the final sample size. LVR was present in 10 (25.0%) subjects. Means and standard deviations of CRP concentrations in the first 50 patients assessed for the overall population and for patients with and without LVR were (1) on admission 2.6 ± 2.2, 2.7 ± 2.8, and 2.6 ± 2.1 mg/L (2) at 24 h after admission 11.5 ± 10.9, 19.4 ± 19.4, and 9.5 ± 6.5 mg/L (3) at discharge 13.0 ± 13.0, 12.3 ± 12.3, and 13.2 ± 13.4 mg/L. Based on these results and assuming a 2-sided alpha value of 0.05, we calculated using the *t*-test for independent variables that enrolment of 195 patients would provide a 99.8% power to demonstrate a significant difference in CRP concentrations at 24 h between patients with and without LVR. We decided to obtain such high power to be able to perform credible multivariate analyses. To compensate for potential withdrawal of consent or loss of study participants due to other reasons, we enrolled 26 additional patients.

The Shapiro-Wilk test demonstrated lack of normal distribution for the majority of the investigated variables. Therefore continuous variables were presented as medians and their interquartile ranges. Depending on the presence or absence of normal distribution, intergroup comparisons were performed with the Student's *t*-test for independent samples or the Mann-Whitney unpaired rank sum test, whereas the Student's *t*-test for paired samples or the Wilcoxon matched-paired rank sum test were applied for comparisons within the groups. Categorical variables were compared using the *χ* ^2^ test with the Yates' correction if required.

Univariate and multivariate logistic regression models were used to identify predictors of LVR. Variables with a *P* value of <0.1 in the univariate analysis and inflammatory markers independently of their *P* values were introduced into the multivariate logistic regression model. Subsequently, variables without any significant impact on the LVR prevalence (*P* ≥ 0.05) were one after another removed from the multivariate model according to their decreasing *P* values. Lastly, to optimize our final multivariate model, we investigated whether addition of variables linked with LVR in previous studies [2, 6, 7, 14] but not in our univariate analysis (time from onset of pain to balloon inflation, LVEF at baseline, wall motion score index at baseline, left ventricular end-systolic volume at baseline, TIMI 3 flow in infract-related artery after pPCI, and presence of multivessel coronary artery disease) would improve its predictive value. Relations between the investigated variables and the likelihood of LVR were estimated with the use of odds ratios (OR) and their 95% confidence intervals (95% CI). The optimal cutoff points were determined using the receiver operator characteristic (ROC) analysis.

The impact of numerous variables upon a quantitative variable was assessed using the multiple regression model.

A two-sided difference was considered significant at *P* < 0.05. The statistical analysis and sample size calculation were carried out using the Statistica 10.0 package (StatSoft, Tulsa, OK, USA), while MedCalc 12.0 (MedCalc Software, Mariakerke, Belgium) statistical software was applied for the ROC analysis.

## 3. Results

### 3.1. Patients

Of the total number of 221 patients enrolled in the study initially, twelve patients were excluded during hospitalization due to false diagnosis of STEMI (3 patients), echocardiographic images insufficient for quantitative analysis (4 patients), expected difficulties in cooperation due to alcohol abuse and dementia (2 patients), dopamine therapy, acute pharyngitis, and abdominal aneurysm rupture (1 patient each). Eleven patients were excluded from further analysis due to incomplete data, including 3 participants who died, 2 with the diagnosis of neoplasmatic disease during followup, and 6 who did not attend the 6-month followup visit. The final study group consisted of 198 patients including 45 (22.7%) women and 153 (77.3%) men (Table 1).

The major adverse cardiac events observed during 6 months of followup in patients with and without LVR included 5 re-MI (2 (3.6%) versus 3 (2.1%); *P* = NS), 24 episodes of unstable angina (7 (12.7%) versus 17 (11.5%) *P* = NS), and 13 episodes of the development of symptomatic heart failure (4 (7.3%) versus 9 (6.3%); *P* = NS). Additionally, within 6 months after the study enrollment 28 patients underwent percutaneous coronary intervention (9 (16.4%) versus 19 (13.3%); *P* = NS) including 8 patients scheduled for an elective procedure during the index hospitalization (2 (3.6%) versus 6 (4.2%); *P* = NS). Furthermore, at 6-month followup, 4 patients had been treated with elective coronary bypass grafting scheduled during the index hospitalization (1 (1.8%) versus 3 (2.1%); *P* = NS).

### 3.2. Clinical, Angiographic, and Echocardiographic Assessment

LVR at 6 months after STEMI was present in 55 (27.8%) patients (Table 1).

In patients with LVR at 6 months after discharge, when compared to those without LV dilatation, anterior location of STEMI, and culprit lesion in the left descending artery were numerically more frequent (Table 1). Similarly, these patients had a higher prevalence of hypertension and diabetes but again the differences were not statistically significant, even though cardiovascular pharmacotherapy at hospital discharge was comparable in both groups (Table 1). We found no differences among the groups in the frequency of angina proceeding STEMI and the diagnosis of heart failure (New York Heart Association class I or II) prior to admission (data not presented).

The LVR group presented with insignificantly longer time from the onset of pain to balloon inflation and noticeably less favourable pre-pPCI angiographic indices of flow in the infarct-related artery (Table 2). However, similar final angiographic results of pPCI were achieved regardless of the future LVR development.

At baseline there were no significant differences in echocardiographic indices between patients with and without LVR at 6 months except for the values of markers of LV diastolic function (Table 3).

At 6-month followup the LVR group had significantly larger diameters of left atrium and left ventricle, higher systolic and diastolic LV volumes, and greater LVM than patients without LV dilatation (Table 3). Similarly, in patients with LVR, we observed significantly higher values of WMSI and markedly lower values of LVEF and average peak systolic mitral annular velocity, indicating more severe impairment of regional, global, and longitudinal LV systolic function (Table 3). Also the echocardiographic indices of LV diastolic function were more abnormal in the LVR group (Table 3).

### 3.3. Biomarkers

Patients who developed LVR, compared to those without LV dilatation, presented with a significantly higher maximal activity of izoenzyme MB of creatine kinase and noticeably increased white blood cell count on admission and at 24 h after admission (Table 2).

**Figure 1:**
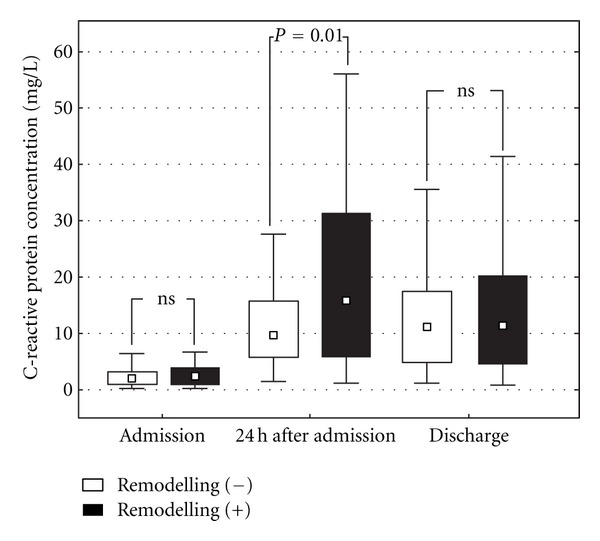
Plasma C-reactive protein concentration on admission, 24 h after admission and at hospital discharge in patients after acute ST-segment elevation myocardial infarction with and without left ventricular remodelling at 6 months after hospital discharge. Results are presented as medians, interquartile ranges, and ranges.

**Figure 2:**
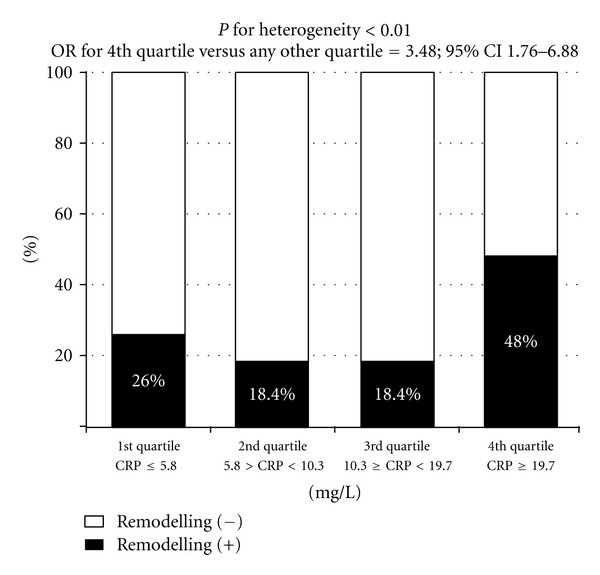
Prevalence of left ventricular remodelling at 6 months after hospital discharge after acute ST-segment elevation myocardial infarction in patients classified according to the increasing quartiles of C-reactive protein concentration 24 h after admission. CRP— C-reactive protein.

Plasma CRP concentration steeply rose during the first 24 h of hospitalization (*P* < 0.001), mainly in the LVR group, and persisted elevated at discharge (Figure 1). CRP concentration 24 h after admission was higher in the LVR group as compared to the patients without LV dilatation at 6 months (15.8 (5.9–31.3) versus 9.5 (5.8–15.6) mg/L; *P* < 0.05). Additionally, as presented in Figure 2, we found a significant heterogeneity of the prevalence of LVR at 6 months among patients classified in quartiles according to CRP concentrations 24 h after admission. The prevalence of LVR at 6 months after STEMI was considerably higher for patients from the highest quartile of CRP concentrations 24 h after admission as compared to those from any other quartile.

BNP concentration increased during hospitalization in all patients (*P* < 0.001). However, its remarkably higher values were observed in the LVR group, mainly at discharge (Table 2).

### 3.4. Predictors of LVR

Predictors of LVR at 6 months after discharge revealed by univariate logistic regression analysis among all demographic, clinical, angiographic, and biochemical variables listed in Tables 1 and 2 as well as among the echocardiographic variables at discharge shown in Table 3 are presented in Table 4. The final model of multivariate logistic regression analysis, including also baseline echocardiographic variables, found high plasma CRP concentration 24 h after admission, elevated BNP plasma concentration at discharge, increased body mass index, and low LVEDV at discharge to be independent predictors of LVR at 6 months after STEMI (Table 4).

When adjusted for plasma CRP concentration, anterior location of STEMI, maximal concentration of troponin I, LVEF at discharge, leukocyte count, and post-PCI flow in infarct-related artery below TIMI 3 grade were no longer associated with the presence of LVR at 6 months in the multivariate analysis.

### 3.5. Optimal Cutoff Values for the Prediction of LVR

A ROC analysis to assess the diagnostic accuracy for the prediction of LVR at 6 months after STEMI revealed areasunderthecurvesof 0.61 (95% confidence interval 0.54–0.68; *P* < 0.05), 0.61 (95% confidence interval 0.54–0.68; *P* < 0.05), 0.59 (95% confidence interval 0.50–0.68; *P* = NS) and 0.53 (95% confidence interval 0.46–0.60; *P* = NS) for plasma CRP concentration 24 h after admission, plasma BNP concentration at discharge, and body mass index and LVEDV at discharge, respectively. The corresponding optimal cutoff values were 14.2 mg/L for CRP 24 h after admission (sensitivity 54.5%, specificity 72.7%, positive predictive value 43.5%, and negative predictive value 80.6%) and 237.3 pg/L for plasma BNP concentration at discharge (sensitivity 40.0%, specificity 82.5%, positive predictive value 46.8%, and negative predictive value 78.1%), respectively. Comparison of theROCcurves demonstrated comparable diagnostic accuracies for both biomarkers (*P* = NS).

### 3.6. Determinants of an Increase in LVEDV

To further investigate the relationship between plasma CRP concentration and the development of LVR, we applied multiple regression analysis to determine which of the demographic, clinical, angiographic, and biochemical parameters listed in Tables 1, 2 and 3 affect an increase in LVEDV between hospital discharge and 6-month followup. High plasma CRP concentration 24 h after admission, elevated BNP values at discharge, and low LVEDV at discharge, but not high body mass index were independently associated with an increase in LVEDV (Table 5).

## 4. Discussion

This study shows the relationship between the inflammatory reaction during STEMI and the development of LVR in long-term followup in patients undergoing pPCI. Moreover, the intensity of early inflammatory response, assessed by plasma CRP concentration measured 24 h after admission to hospital, was found to be an independent predictor of LVR at 6 months after a first STEMI. Furthermore, the robustness of our findings was confirmed in a multiple regression analysis which indicated the presence of an independent association between incremental plasma CRP concentrations and an increase in LVEDV, a marker of LVR. On the other hand, the ROC analysis revealed a limited discriminative value of CRP assessment in terms of the postinfarct LVR prediction.

To our best knowledge, it is the first adequately powered study linking in-hospital plasma CRP concentration and postinfarct LVR at 6 months after discharge, conducted exclusively in a STEMI population undergoing pPCI. In a small study (*n* = 48) by Mather et al., CRP level measured 2 days after reperfusion was found to be a predictor of LVR at 3 months [20]. Similarly, Ørn et al. found in 42 patients undergoing pPCI a significant correlation between plasma CRP concentration assessed 2 days after the intervention and the parameters of LVR at 2 months after STEMI [15]. In accordance with these studies Fertin et al. demonstrated a relationship between in-hospital CRP level and LVR in long-term followup in 226 patients with a first anterior Q-wave MI, but in apparent contrast to our study, CRP concentration was not independently associated with LVR [14]. This discrepancy among the trials may be related to differences in the sample sizes, heterogeneity of study populations, modalities of treatment and timing of blood sampling. The greater prevalence of LVR in the study by Fertin et al. (38%) as compared to previous and our studies (28–30%) may be caused by exclusive inclusion of patients with anterior MI, only 52% of whom underwent pPCI [14].

It should be acknowledged that a single blood sampling for plasma CRP concentration measure drawn only at hospital discharge (i.e., when the intensity of the inflammatory reaction is already decreasing) may also provide different results [14]. Multiple blood sampling throughout the hospital period to obtain CRP peak values employed in our study revealed lack of difference in plasma CRP concentration at discharge between the LVR and non-LVR groups. Thus, determination of CRP level at this time point is unlikely to improve the accuracy of LVR prediction. It should be emphasized that our population of patients was homogenous, with strictly applied criteria of inclusion and exclusion. In our opinion, this, together with the delivery of unison invasive and pharmacologic treatment, allowed us to minimise the risk for confounding factors and enabled to reliably quantify the relationship between plasma CRP concentration and the development of postinfarct LVR.

Our study confirms previous evidence of the presence of a significant increase in CRP concentration in the course of MI [8, 10, 15]. As reported by several studies, elevated CRP levels after MI are associated with adverse clinical outcome, including cardiac rupture, heart failure, and cardiac death [9, 12, 13]. Myocardial necrosis due to abrupt closure of coronary artery, in case of acute MI, leads to a systemic and regional humoral and cellular inflammatory response aiming to promote the local myocardial healing process and scar formation [21, 22]. In the early phase of MI, cytokines play an important cytoprotective role, mainly by reducing cell apoptosis [10]. However, in case of exuberant inflammatory reaction, high tissue cytokine levels may persist for a long time and the extent of the primary ischaemically damaged myocardial tissue may paradoxically increase [21]. Plasma CRP concentration increases following the cytokines activation in the first hours of MI [8, 15]. The rapid and sustained rise in plasma CRP concentration, in the early phase of hospitalization in patients with LVR seen in our study, reflects the severity of the inflammatory reaction within the infarcted area in patients with STEMI. CRP binds to phosphocholine groups of necrotic myocardial cell membranes, facilitating complement activation, and thus promoting further inflammatory response, injury of myocardial cells, and expansion of necrosis [23–26]. An experimental study by Takahashi et al. has clearly demonstrated that increased CRP expression exacerbates LV dysfunction and promotes LVR after MI [11]. Literature data also suggests that not only is CRP an inflammatory marker, but it should also be considered as an inflammatory mediator holding prothrombotic and proapoptotic properties [23–26]. There is evidence that apoptosis, both at the site of infarction and in unaffected regions of LV, correlates with unfavourable postinfarct LVR and the occurrence of heart failure after MI [27]. The size of the healed infarction has been demonstrated to be a major determinant of long-term LVR and a stronger predictor of all-cause mortality than LVEF [28]. The deleterious effect of CRP on postinfarct LVR may be independent of myocardial necrosis size and may be associated with increased apoptotic rates, macrophage infiltration, monocyte chemotactic protein expression-1, and matrix metalloproteinase-9 activity in the border zone [11]. 

It is currently known that a strong correlation exists between CRP levels, infarct size, and biochemical markers of myocardial necrosis [15, 29, 30]. The findings by Ørn provide a potential pathophysiological explanation for the association between CRP levels and LVR, by linking peak CRP levels with infarct size after 2 months [15]. Also in our study including patients with a first STEMI undergoing pPCI, the LVR group exhibiting a more pronounced inflammatory response, presented with larger infarctions as assessed by enzymatic assays, mainly of anterior location. The sustained increase in plasma CRP concentration in the course of MI observed in the group of patients with LV dilatation may have been associated with a greater extent of myocardial damage reflected by the values of cardiac necrosis markers. In contrast, Haase et al. did not confirm this observation possibly due to different clinical characteristics of the study population admitted for rescue coronary angioplasty, 30% of whom had a previous MI that might have precluded accurate assessment of new myocardial damage [31]. 

We also found one of the markers of STEMI-induced LV haemodynamic impairment in the course of STEMI (i.e., BNP plasma level at discharge) to be an important independent predictor of cardiac dilatation after discharge. When compared with the no LVR group, patients with LVR present at 6-month followup, not only showed higher initial BNP levels, but also their persistence during hospitalization. Evaluation of BNP has been known as a useful tool for detecting LV dysfunction and as a risk marker for adverse clinical outcome in patients with STEMI [32]. Cardiac natriuretic peptides reflect the ventricular function impairment and haemodynamic decompensation in the course of MI. According to our results, they could also be useful for predicting LVR following STEMI since they take into account not only the loss of necrotic myocardium, infarct expansion, and increased LV wall stress but also the effects of compensatory reaction developing during early stages of LV remodelling for example, increased contractility of the intact muscle [33, 34]. The inclusion of both biomarkers (CRP and BNP) into our analysis might have improved risk prediction as these biomarkers provide additive prognostic information in patients with acute coronary syndromes [35]. However, as reported by Fertin in a study including patients with a first anterior STEMI, despite existing correlations between CRP and BNP and long-term LVR, none of these biomarkers at discharge was independently predictive of LVR at 12 months [14].

Noteworthy, we also demonstrated a link between low LVEDV at discharge and the development of LVR in long-term followup. This somewhat unexpected observation is in line with previous findings [2, 36]. Interpreting these results Bolognese et al. hypothesize that LVR predominantly occurs in patients with large functional infarct size that initially does not compromise the overall LV cavity dimensions and function [2].

Furthermore, in our study overweight and obese patients were more likely to present with LVR at 6 months. High body mass index is linked with both proinflammatory state and a rise in total blood volume resulting in increased cardiac output [37]. Obesity subsequently leads to LV dilation, increased LV wall stress, eccentric LV hypertrophy, and LV diastolic dysfunction [37]. Our results correspond with previous observations from a mice model of MI that indicated in diet-induced obese animals, when compared to controls, a significant reduction in collagen deposition within the scar along with a more pronounced LV dilation and cardiac hypertrophy, indicative of adverse LVR [38].

## 5. Limitations of the Study

Although our study throws some new light on the mechanisms of LVR development in patients treated with pPCI for their first STEMI, it has several limitations. First, it lacks adequate power to assess clinical end points. The low rate of clinical events observed in our patients is driven by high efficacy of modern MI management, medium-term followup and application of strict inclusion and exclusion criteria. Second, due to early achievement of reperfusion the patients had relatively well-preserved LV function. Third, we did not account in our calculations for diurnal and seasonal variations in CRP concentration. Fourth, further efforts are warranted to confirm the clinical significance of our findings and to fully explain the mechanisms through which augmentation of the inflammatory process contributes to the occurrence of LVR. Fifth, we did not assess subpopulations of leukocytes but only their overall count which precludes any investigation of the potential relationship between leukocyte types, CRP concentration, and the development of LVR. Sixth, the enrolment of participants to our study was completed four years ago but this fact seems to have no major impact on the value of obtained results as STEMI management has not been markedly changed since then. Finally, as the LVR and non-LVR groups differed in several aspects, it cannot be fully excluded that the higher incidence of LVR and the modest difference in CRP elevations may be related to the fact that the LVR-group altogether had a more frequent anterior location of STEMI as well as a higher body mass index and prevalence of both hypertension and diabetes. However, differences for each of these potential confounders assessed separately were statistically insignificant between the LVR and non-LVR groups.

## 6. Conclusions

The measurement of CRP plasma concentration at 24 h after admission possesses a statistically significant but modest value in the prediction of long-term LVR in patients after a first STEMI treated by pPCI.

## Figures and Tables

**Table 1:** Demographic and clinical characteristics of the study population.

Variable	Overall study population (*n* = 198)	Patients with LVR (*n* = 55)	Patients without LVR (*n* = 143)	*P* for comparison between groups with and without LVR
Age (years)	56.0 (50.0–64.0)	54.0 (49.0–63.0)	57.0 (51.0–64.0)	NS
Gender (male/female) *n* (%)	153/45 (77.3/22.7)	46/9 (83.6/16.4)	107/36 (74.8/25.2)	NS
Anterior location of STEMI *n* (%)	89 (43.6)	30 (54.6)	58 (40.6)	NS
Time from onset of pain to balloon inflation (minutes)	201.5 (141.0–295.0)	207.0 (135.0–308.0)	200.0 (145.0–295.0)	NS

Risk factors for coronary artery disease				

Body mass index (kg/m^2^)	26.8 (24.2–29.1)	27.5 (24.8–30.4)	26.2 (24.1–28.8)	NS
Hypertension *n* (%)	82 (41.4)	27 (49.1)	55 (38.5)	NS
Diabetes mellitus *n* (%)	40 (20.2)	13 (23.6)	27 (18.9)	NS
Current or ex-smoker *n* (%)	131 (66.2)	34 (61.8)	97 (67.8)	NS

Medical treatment at hospital discharge				

Long-acting metoprolol *n* (%)	196 (99.0)	55 (100.0)	141 (98.6)	NS
Perindopril *n* (%)	194 (98.0)	53 (96.4)	141 (98.6)	NS
Simvastatin *n* (%)	197 (99.5)	55 (100.0)	142 (99.3)	NS
Spironolactone *n* (%)	12 (6.1)	5 (9.1)	7 (4.9)	NS
Nonpotassium-sparing diuretics *n* (%)	11 (5.6)	4 (7.3)	7 (4.9)	NS

LVR: left ventricular remodelling; STEMI: ST-segment elevation myocardial infarction.

**Table 2:** Angiographic and biochemical characteristics of the study population.

Variable	Overall study population (*n* = 198)	Patients with LVR (*n* = 55)	Patients without LVR (*n* = 143)	*P* for comparison between groups with and without LVR
Angiographic indices				

IRA: LAD/non-LAD *n* (%)	92 (46.5)/106 (53.5)	32 (58.2)/23 (41.8)	60 (42.0)/83 (58.0)	NS
Multivessel coronary artery disease *n* (%)	119 (60.0%)	38 (69.1%)	81 (56.6%)	NS
TIMI 3 flow in IRA *n* (%)				
Before pPCI	55 (27.8)	10 (18.2)	45 (31.5)	<0.05
After pPCI	185 (93.4)	49 (89.1)	136 (95.1)	NS
TMPG 3 after pPCI *n* (%)	94 (46.1)	30 (54.6)	62 (43.4)	NS
Patients with implanted stents *n* (%)	196 (99.0)	54 (98.2)	142 (99.3)	NS
Abciximab use *n* (%)	49 (24.7)	17 (30.9)	32 (22.4)	NS

Biochemical parameters				

Creatinine (*μ*mol/L)	82.7 (72.0–95.9)	82.7 (79.6–89.3)	82.2 (70.7–97.2)	NS
Admission glucose (mmol/L)	7.6 (6.8–9.3)	7.8 (6.8–9.6)	7.4 (6.7–9.2)	NS
TnI_max_ (ng/mL)	46.3 (11.7–50.0)	50.0 (15.3–50.0)	36.7 (10.5–50.0)	NS
CK-MB_max_ (U/L)	102.5 (58.0–160.0)	134.0 (87.0–206.0)	95.0 (54.0–141.0)	<0.01
LDL cholesterol (mmol/L)	3.76 (3.23–4.45)	3.75 (3.39–4.40)	3.78 (3.21–4.45)	NS
HDL cholesterol (mmol/L)	1.32 (1.16–1.50)	1.32 (1.19–1.50)	1.34 (1.14–1.50)	NS
Triglycerides (mmol/L)	1.01 (0.67–1.59)	0.98 (0.64–1.51)	1.02 (0.69–1.59)	NS
Leukocyte count on admission (10^3^/*μ*L)	11.3 (9.1–13.3)	12.0 (10.5–13.7)	10.6 (8.7–12.8)	<0.05
Leukocyte count 24 h after admission (10^3^/*μ*L)	10.1 (8.6–11.7)	10.3 (9.2–12.3)	10.1 (8.2–11.3)	<0.05
BNP on admission (pg/mL)	51.9 (25.9–102.5)	50.5 (25.3–121.0)	52.8 (26.8–93.0)	NS
BNP at discharge (pg/mL)	124.8 (70.5–233.0)	159.6 (74.6–358.2)	114.0 (61.5–192.7)	<0.05

BNP: B-type natriuretic peptide; CK-MB_max_: maximal activity of izoenzyme MB of creatine kinase; HDL cholesterol: high-density-lipoprotein cholesterol; IRA: infarct-related artery; LAD: left anterior descending artery; LDL cholesterol: low-density-lipoprotein cholesterol; LVR: left ventricular remodelling; pPCI: primary percutaneous coronary intervention; TnI_max_: maximal concentration of troponin I; TIMI: Thrombolysis in Myocardial Infarction score; TMPG: TIMI myocardial perfusion grade.

**Table 3:** Echocardiographic characteristics of the study population. Echocardiographic indices are derived from 2D, Doppler echocardiography, and tissue Doppler echocardiography at hospital discharge and at 6 month after discharge, respectively.

Variable	Overall study population (*n* = 198)	Patients with LVR (*n* = 55)	Patients without LVR (*n* = 143)	*P* for comparison between groups with and without LVR
Echocardiographic indices at discharge				

LA (mm)	39.0 (36.0–42.0)	39.0 (36.0–45.0)	39.0 (36.0–41.0)	NS
LVEDd (mm)	49.0 (45.0–53.0)	49.0 (43.0–53.0)	49.0 (45.0–53.0)	NS
LVESd (mm)	34.0 (30.0–37.0)	34.0 (30.0–38.0)	33.0 (30.0–36.0)	NS
LVMI (g/m²)	114.3 (96.7–132.3)	114.4 (99.0–133.4)	114.1 (96.5–132.3)	NS
LVEDV (mL)	97.0 (82.0–118.0)	95.0 (81.0–118.0)	99.0 (83.0–118.0)	NS
LVEDVI (mL/m²)	50.5 (44.0–60.8)	48.7 (42.0–57.9)	51.4 (45.6–62.1)	NS
LVESV (mL)	53.5 (44.0–68.0)	57.0 (43.0–66.0)	52.0 (44.0–68.0)	NS
LVESVI (mL/m²)	27.7 (23.1–35.0)	26.9 (23.0–34.1)	27.7 (23.2–35.7)	NS
LVEF (%)	44.8 (40.0–49.4)	43.0 (38.5–48.0)	45.0 (40.4–49.9)	NS
WMSI (pts)	1.5 (1.38–1.75)	1.63 (1.38–1.75)	1.5 (1.38–1.75)	NS
S′ (cm/s)	7.01 (6.14–8.15)	6.75 (5.98–7.9)	7.15 (6.3–8.15)	NS
*E*/*A*	0.84 (0.66–1.09)	0.85 (0.68–1.12)	0.82 (0.64–1.06)	<0.05
DT (ms)	155.0 (145.0–185.0)	150.0 (140.0–170.0)	160.0 (150.0–190.0)	<0.001
*E*/*E*′	9.6 (7.3–11.8)	9.9 (5.3–20.6)	9.5 (7.3–11.5)	NS

Echocardiographic indices at 6 month after discharge				

LA (mm)	40.0 (37.0–44.0)	42.0 (38.0–46.0)	40.0 (37.0–43.0)	<0.05
LVEDd (mm)	50.0 (46.0–54.0)	53.0 (47.0–56.0)	49.0 (46.0–53.0)	<0.01
LVESd (mm)	34.0 (31.0–37.0)	36.0 (32.0–40.0)	33.0 (31.0–36.0)	<0.01
LVMI (g/m²)	115.3 (102.7–134.8)	124.5 (108.3–143.0)	112.1 (98.9–130.3)	<0.01
LVEDV (mL)	110.0 (91.0–133.0)	128.0 (110.0–154.0)	100.0 (88.0–126.0)	<0.001
LVEDVI (mL/m²)	57.2 (48.8–68.3)	66.6 (55.2–86.0)	54.0 (46.5–65.3)	<0.001
LVESV (mL)	57.0 (46.0–74.0)	72.0 (55.5–94.0)	51.2 (43.0–68.0)	<0.001
LVESVI (mL/m²)	29.2 (24.8–38.9)	35.3 (27.8–46.1)	27.7 (23.5–35.9)	<0.001
LVEF (%)	46.0 (42.3–52.0)	44.0 (38.0–49.0)	46.6 (43.0–52.0)	<0.01
WMSI (pts)	1.44 (1.31–1.63)	1.5 (1.38–1.75)	1.44 (1.31–1.63)	<0.01
S′ (cm/s)	6.98 (6.13–8.13)	6.5 (5.55–7.58)	7.08 (6.23–8.28)	<0.01
*E*/*A*	0.85 (0.67–1.12)	0.89 (0.75–1.37)	0.82 (0.64–1.06)	<0.01
DT (ms)	170.0 (155.0–195.0)	160.0 (150.0–185.0)	179.0 (155.0–200.0)	<0.05
*E*/*E*′	8.9 (7.5–11.1)	9.7 (5.7–22.4)	8.7 (7.2–10.1)	<0.05

DT: Deceleration time of early transmitral flow; *E*: peak velocity of early transmitral flow; *A*: peak velocity of transmitral flow during atrial systole; *E*′: average peak early diastolic mitral annular velocity; LA: left atrium; LVEDd: left ventricular end-diastolic diameter; LVEDV: left ventricular end-diastolic volume; LVEDVI: left ventricular end-diastolic volume index; LVEF: left ventricular ejection fraction; LVESd: left ventricular end-systolic diameter; LVESV: left ventricular end-systolic volume; LVESVI: left ventricular end-systolic volume index; LVMI: left ventricle mass index; LVR: left ventricular remodelling; *S*′: average peak systolic mitral annular velocity; WMSI: wall motion score index.

**Table 4:** Predictors of postinfarct left ventricular remodelling in univariate and multivariate analyses. Univariate analysis shows demographic, clinical, angiographic, biochemical, and discharge echocardiographic parameters from Tables 1–3 with a *P* value <0.1 as well as BNP, CRP, and leukocyte count independently of their *P* values, while results of multivariate analysis are restricted to variables with a *P* value <0.05. Results are presented according to decreasing values of odds ratios.

Univariate analysis			
	OR	95% CI	*P*
CRP on admission (for a 10 mg/L increase)	2.99	0.60–14.92	NS
IRA: LAD versus non-LAD	1.92	1.02–3.63	<0.05
Anterior versus nonanterior wall STEMI	1.76	0.94–3.30	0.077
Diabetes mellitus	1.46	0.95–2.24	0.086
CRP 24 h after admission (for a 10 mg/L increase)	1.29	1.08–1.56	<0.01
TnI_max_ (for a 10 ng/mL increase)	1.18	0.99–1.40	0.051
BNP at discharge (for a 100 pg/mL increase)	1.14	1.02–1.28	<0.05
Body mass index (for a 1 kg/m^2^ increase)	1.10	1.01–1.20	<0.05
Leukocyte count on admission (for a 10^3^/*μ*L increase)	1.10	0.99–1.22	0.068
Leukocyte count 24 h after admission (for a 10^3^/*μ*L increase)	1.09	0.97–1.23	NS
BNP on admission (for a 100 pg/mL increase)	1.08	0.88–1.33	NS
CK-MB_max_ (for a 10 U/L increase)	1.07	1.03–1.11	<0.001
CRP at discharge (for a 10 mg/L increase)	1.05	0.86–1.30	NS

Multivariate analysis			

CRP 24 h after admission (for a 10 mg/L increase)	1.29	1.04–1.60	<0.05
BNP at discharge (for a 100 pg/mL increase)	1.21	1.05–1.39	<0.01
Body mass index (for a 1 kg/m^2^ increase)	1.10	1.01–1.21	<0.05
LVEDV at discharge (for a 1 mL increase)	0.98	0.96–0.99	<0.01

BNP: B-type natriuretic peptide; CI: confidence interval; CK-MB_max_: maximal activity of izoenzyme MB of creatine kinase; CRP: C-reactive protein; IRA: infarct-related artery; LAD: left anterior descending artery; LVEDV: left ventricular end-diastolic volume; MI: myocardial infarction; NYHA: New York Heart Association; OR: odds ratio; STEMI: ST-segment elevation myocardial infarction; TnI_max_: maximal concentration of troponin I.

**Table 5:** Impact of demographic, clinical, angiographic, and biochemical variables from Tables 1, 2, and 3 on an increase in end-diastolic left ventricular volume between hospital discharge and 6-month followup.

Model characteristics: *R* = 0.38; *R* ^2^ = 0.13; *P* < 0.00001					
	Beta coefficient	Beta coefficient standard error	Direction component beta	Direction component beta standard error	*P*
Intercept			28.94	4.75	<0.0001
CRP 24 h after admission (for a 10 mg/L increase)	0.21	0.07	2.08	0.72	<0.005
LVEDV at discharge (for a 1 mL increase)	−0.32	0.07	−0.22	0.05	<0.0001
BNP at discharge (for a 100 pg/mL increase)	0.25	0.07	1.64	0.48	<0.0008

BNP: B-type natriuretic peptide; CRP: C-reactive protein; LVEDV: end-diastolic left ventricular volume.
